# Single-Nucleotide Polymorphisms Within MicroRNAs Sequences and Their 3' UTR Target Sites May Regulate Gene Expression in Gastrointestinal Tract Cancers

**DOI:** 10.5812/ircmj.16659

**Published:** 2014-07-05

**Authors:** Zahra Saadatian, Andrea Masotti, Ziba Nariman Saleh Fam, Behnam Alipoor, Milad Bastami, Hamid Ghaedi

**Affiliations:** 1 Medical Genetics Department, Faculty of Medicine, Tabriz University of Medical Sciences, Tabriz, IR Iran; 2 Gene Expression-Microarrays Laboratory, IRCCS Bambino Gesu Children's Hospital, Rome, Italy; 3 Medical Genetics Department, Faculty of Medicine, Tehran University of Medical Sciences, Tehran, IR Iran; 4 Clinical Biochemistry Department, Faculty of Medicine, Tehran University of Medical Sciences, Tehran, IR Iran; 5 Medical Genetics Department, Faculty of Medicine, Shahid Beheshti University of Medical Sciences, Tehran, IR Iran; 6 Genomic Research Center, Taleghani Hospital, Shahid Beheshti University of Medical Sciences, Tehran, IR Iran

**Keywords:** MicroRNAs, Gastrointestinal Neoplasms, Colorectal Neoplasms, Gastric Neoplasms, Esophageal Neoplasms, Single Nucleotide Polymorphisms

## Abstract

**Background:**

Esophageal, stomach, and colorectal cancers are commonly lethal gastrointestinal tract (GIT) neoplasms, causing almost two million deaths worldwide each year. some environmental risk factors are acknowledged; however, genetic defects can significantly contribute to predisposition to GIT cancers. Accordingly, recent works have shown that single-nucleotide polymorphisms (SNPs) within miRNAs coding sequence (miR-SNPs) and miRNA target sites (target-SNPs) may further contribute to increased risk of developing cancer.

**Objectives:**

In this study, we comprehensively identified miRNA-target gene pairs implicated in GIT cancers and catalogued the presence of potentially functional miR-SNPs and target-SNPs that impair the correct functional recognition.

**Materials and Methods:**

Using bioinformatics tools, manual literature review, and a highly accurate dataset of experimentally validated miRNA-target gene interactions, we compiled a list of miRNA-target genes pairs related to GIT cancers and prioritized them into different groups based on the levels of experimental support. Functional annotations (gene ontology) were applied to these pairs in each group to gain further information.

**Results:**

We identified 97 pairs in which both miRNAs and target genes were implicated in GIT cancers. Several pairs, denoted as highly polymorphic pairs, had both miR-SNPs and target-SNPs. In addition, more than 5000 miRNA-target gene pairs were identified in which, according to the previous reports, either the miRNAs or the target genes had a direct involvement in GIT cancers. More than 800 target-SNPs are located in regulatory regions that were extracted from the ENCODE project through the RegulomeDB database. Of these, 20 were classified as expression quantitative trait loci (eQTLs).

**Conclusions:**

Our work provided a comprehensive source of prioritized and annotated candidate polymorphisms inside miRNAs and their target sites in GIT cancers, which would facilitate the process of choosing right candidate miRNA-target genes and related polymorphisms for future association or functional studies.

## 1. Background

Esophageal, stomach, and colorectal cancers are commonly lethal gastrointestinal tract (GIT) malignancies, causing annually more than 1753000 deaths worldwide (1). Esophageal cancer (EC) is the eighth most common cancer and the sixth leading cause of cancer mortality, with a five-year overall survival rate of 10% to 16% (2, 3). Gastric cancer (GC), the fourth most common tumor and the second leading cause of mortality, is often diagnosed in advanced ages and has an average survival rate of only seven to nine months (4-6). Colorectal cancer (CRC), the third most common cancer in men and the second one in women, is responsible for approximately 8% of all cancer deaths (7). Despite some well acknowledged environmental risk factors, genetic defects can significantly contribute to predisposition to GIT cancers. MicroRNAs (miRNAs) are a class of small noncoding RNAs that are evolutionary conserved and involved in posttranscriptional regulation of gene expression (8). They modulate gene expression by binding to 3' UTR target sites of mRNAs and repress their translation or promote cleavage and degradation (9). MiRNAs regulate nearly all cellular processes that are altered during tumorigenesis; their widespread contribution to cancer has been investigated (10). Researchers have obtained an overwhelming amount of data suggesting that single-nucleotide polymorphisms (SNPs) in miRNAs (miR-SNPs) and their target sites (target-SNPs) may be associated with an altered risk of developing cancer (9).

## 2. Objectives

In this work, we comprehensively identified and catalogued miRNA-target gene pairs in GIT cancers and annotated relevant candidate miR-SNPs and their target-SNPs in order to study the potential implications of SNPs in the developing GIT cancers.

## 3. Materials and Methods

We considered esophageal, stomach, and colorectal cancers as representative GIT tumors. Using an in silico approach, we extracted information on SNPs in GIT cancer-related miRNA-target mRNAs. First, we compiled information on the miRNA-target mRNA duplexes with some evidence of contribution into GIT cancers by searching relevant papers in the literature. To this aim, we assembled two lists: one for coding genes and another for miRNAs implicated in GIT cancers; we called them GI-genes (GIT cancers-related genes) and GI-miRNAs lists, respectively. Then, we employed a data set of experimentally validated miRNA-target gene pairs to retain all of the GIT cancer-relevant pairs. Furthermore, we categorized the identified miRNA-target gene pairs into three groups (A, B, and C). Whenever a miRNA-target gene pair was included in GI-miRNAs or GI-genes lists, we assigned the pair to the group-A. Group-B included hosts pairs in which only miRNAs were retrieved from GI-miRNAs list, i.e. target genes were not from GI-gene list. Group-C consisted of pairs in which miRNAs were not from GI-miRNAs list but their target genes belonged to the GI-gene list. Next, we annotated miRNAs-target gene pairs in all of these groups and extracted information about the presence of SNPs in miRNA sequence (miR-SNPs) and in the 3' UTR mRNA sequence (target-SNPs) by using different bioinformatics resources (Table 1). What follows in the remaining of this section is a more detailed description of data sets, resources, and procedures employed in this study.

**Table 1.** URLs of Bioinformatics Tools Employed in This Study ^a^

Tools	URLs	Brief Description
**miRBase**	http://www.mirbase.org/	A searchable database of published miRNA sequences and annotation
**TUMIR**	http://www.ncrnalab.com/TUMIR/	An experimentally supported database of miRNA deregulation in various cancers
**CAMi-Finder**	http://www.isical.ac.in/~bioinfo_miu/web_isi.html	A Cancer Associated MicroRNA Mining Tool
**miR2Disease**	http://www.mir2disease.org/	A comprehensive resource of miRNA deregulation in various human diseases
**HuGE Navigator**	http://hugenavigator.net/HuGENavigator/home.do	A knowledge base in human genome epidemiology, including genetic association
**CancerGAMAdb**	http://www.hugenavigator.net/CancerGEMKB/caIntegratorStartPage.do	A continually updated database of genetic association studies in cancer
**Cancer Gene Census**	http://cancer.sanger.ac.uk/cancergenome/projects/census/	A catalog of those genes for which mutations have been causally implicated in cancer
**Network of Cancer Genes (NCG4)**	http://bio.ieo.eu/ncg/	Reports annotation on 2000 protein-coding cancer genes
**DAVID**	http://david.abcc.ncifcrf.gov/	A comprehensive set of functional annotation tools
**Gene Expression Atlas**	http://www.ebi.ac.uk/gxa/	Provides information on gene expression patterns under different biological conditions
**dbGaP**	http://www.ncbi.nlm.nih.gov/sites/entrez?db=gap	An archive of the results of studies investigating interaction of genotypes and phenotypes
**NHGRI GWAS catalog**	www.genome.gov/gwastudies	A Catalog of Published Genome-Wide Association Studies
**RegulomeDB**	http://www.regulomedb.org/	A database that annotates SNPs with known and predicted regulatory elements
**PolymiRTS 3.0**	http://www.regulomedb.org/	A database of SNPs in microRNAs and their target sites

^a^ Abbreviations: SNP, single-nucleotide polymorphisms; and mi RNA, microRNA.

### 3.1. Gastrointestinal Tract Cancers-Related Genes 

This list contained GI-genes extracted from the Cancer Gene Census (11), candidate genes extracted from the Cancer Gene Network (version 4) (12), which is a collection of manually curated genes from 77 whole genome or whole exome cancer resequencing experiments, and candidate genes from Cancer Genome-wide Association and Meta-analysis Database (CancerGAMAdb), which is a database of Genome-wide Association Studies (GWAS) and meta-analysis data in cancer (13).

### 3.2. Gastrointestinal Tract Cancers-Related MicroRNAs 

In the GI-miRNAs list, we included miRNA with at least one of the following essential characteristics:

- Altered expression in GIT cancers.
- Evidence for association of a miRNA-hosted-SNP with GIT cancers or their outcome.

Data for altered expression of miRNAs were retrieved from TUMIR (14), CAMi-Finder, miR2 disease (15), and from the literature (2, 4, 7, 16, 17). For associations, we used HuGE Navigator (last updated on November 29, 2013), an integrated database of genetic associations (13). MiRNA coordinates were retrieved from miRBase (release 20) (18, 19).

### 3.3. MicroRNA-Target Gene Interactions

Recently, a technique, named CLASH (crosslinking, ligation, and sequencing of hybrids), has been developed for ligation and sequencing of miRNA-target RNA duplexes associated with human AGO1 (20). Using CLASH, Helwak et al. have reported a data set of more than 18000 high-confidence miRNA-mRNA interactions, which represents a breakthrough in the field (20). Therefore, we took advantage of CLASH datasets as a source of experimentally validated miRNA-target interactions. The coordinates of SNPs in binding sites where retrieved from PolymiRTS 3.0 database (21).

### 3.4. Functional Annotation Analysis

Gene list functional analysis was performed by the Database for Annotation, Visualization and Integrated Discovery (DAVID v. 6.7) with the EASE score threshold, i.e. P Value, being 0.05 and count threshold, i.e. minimum number of genes for corresponding term being set at two (22, 23).

### 3.5. Differential Gene Expression Data Sets

Gene Expression Atlas was searched for upregulated or downregulated genes in GIT tumors by searching terms such as colon cancer, colorectal cancer, gastric cancer, and esophageal cancers (24). The differential expression of genes reported in four previous studies (experiment accession IDs: E-GEOD-13471, E-GEOD-1420, E-GEOD-19249, E-GEOD-19826, E-GEOD-23878, E-GEOD-2685, E-MTAB-57, E-MTAB-62, and E-MTAB-145) were downloaded and employed in this work (25-33).

### 3.6. The Encyclopedia of DNA Elements and Expression Quantitative Trait Loci Data

RegulomeDB is a database that annotates SNPs with known and predicted regulatory elements in the intergenic regions of the Homo sapiens genome. These elements include regions of DNAase hypersensitivity, binding sites of transcription factors, and promoter regions. The source of these data includes public datasets from GEO, the Encyclopedia of DNA Elements (ENCODE) project, and published literature (34). We employed this database to find target-SNPs located in annotated regions and test their function as expression quantitative trait loci (eQTLs).

## 4. Results

### 4.1. Group-A (GI-miRNA:GI-Gene Pairs)

We identified a total of 36 GI-miRNAs, altogether regulating 66 GI-genes in the form of 97 unique miRNA-target duplexes. Searching for SNPs inside miRNAs sequences and mRNA-binding sites in this group, we identified 29 miR-SNPs (25 with frequency information) and 150 target-SNPs (61 with frequency information). Among these, we looked for "highly polymorphic pairs", which bore both miR-SNP and target-SNP. We found several notable examples, including *hsa-miR-93-5p:BIRC5* and *hsa-miR-149-5p:BIRC5*. Survivin gene (*BIRC5*) belongs to inhibitor of apoptosis gene family and is implicated in GIT cancers. On the other hand, both regulators of survivin (*hsa-miR-93-5p and hsa-miR-149-5p*) are also involved in GC. In the highly polymorphic pairs we identified *hsa-miR-196a-5p *and* hsa-miR-92-3p*. The first one seems to be involved in all GIT cancer types, and the second has a crucial role in CRC and GC, by regulating 20 GI-genes, 14 of which had SNPs in binding sites (Table 2). In addition, *hsa-miR-222-3p* and *hsa-miR-100-5p* were involved in two GIT cancers (CRC/GC and CRC/EC respectively).

**Table 2.** MicroRNAs in Highly Polymorphic Pairs ^a,b^

MiRNA	MiRNA Alteration in GIT Cancers	MiR-SNP	Number of Targets With Target-SNPs	Number of Target-SNPs	GIT Cancer
***hsa-miR-148b-3p***	CRC (down)	rs74878365	2	2	CRC
***hsa-miR-149-5p***	GC (association)	rs2292832; rs71428439	2	3	GC, EC, and CRC
***hsa-miR-15b-5p***	GC (up)	rs146020563; rs192595529	1	1	GC
***hsa-miR-16-5p***	CRC (up)	rs72631826	3	4	CRC
***hsa-miR-182-5p***	CRC (up)	rs76481776; rs77586312; rs80041074	1	1	CRC
***hsa-miR-194-5p***	CRC (down)	rs11231898	1	2	CRC
***hsa-miR-196a-5p***	GC and EC (up); CRC, GC, and EC (association)	rs11614913; rs185070757; rs186449583; rs190478598;	2	4	GC, EC, and CRC
***hsa-miR-20a-5p***	CRC (up)	rs185831554	1	2	CRC
***hsa-miR-222-3p***	CRC and GC (up)	rs187612279; rs191727254; rs72631825	3	9	CRC and GC
***hsa-miR-26a-5p***	CRC (association)	rs182070256; rs190898565	3	4	CRC and GC
***hsa-miR-30a-3p***	CRC (down)	rs149150037; rs190842689	1	2	CRC
***hsa-miR-30b-5p***	GC (up)	rs111424617	1	2	GC
***hsa-miR-605-5p***	CRC (association)	rs113212828; rs2043556	1	3	CRC
***hsa-miR-92a-3p***	CRC (up/down) and GC (up)	rs9589207	14	42	CRC and GC
***hsa-miR-93-5p***	GC (up)	rs72631824	1	2	GC, EC, and CRC

^a^ Abbreviations: CRC, colorectal cancer; Down, downregulated; EC, esophageal cancer; GC, gastric cancer; and Up, upregulated.

^b^ The table shows miRNA of each highly polymorphic pair, its alteration in cancer and relevant pre-miRNA SNPs, the number of its target with SNPs in their binding sites, and the total number of SNPs in these binding sites. The last column shows involvement of target genes bearing target-SNPs in GIT cancers.

### 4.2. Group-B (GI-miRNA:Non-GI-Genes)

This group comprises 7763 interactions including 83 unique GI-miRNAs and 4549 unique non-GI-target genes. We tested the hypothesis that some target genes in group-B might have functional relevance to GIT cancers. By performing functional annotation analysis to the GI-genes list and group-B target genes, we found that about 64% of targets in group-B were involved in the same biological processes as GI-genes. These genes were kept in the list of potential candidate genes. The five most enriched processes in GI-genes list were positive regulation of cell differentiation (GO: 0045597), enzyme linked receptor protein signaling pathway (GO: 0007167), regulation of programmed cell death (GO: 0043067), phosphate metabolic process (GO: 0006796), and cell surface receptor-linked signal transduction (GO: 0007166). To further support these evidences, we explored differential gene expression data sets of Gene Expression Atlas to identify upregulated or downregulated genes in GIT tumor in comparison with normal tissues. Intersection analysis of differentially expressed genes and potential candidate genes indicated that more than 94% (2745 of 2904) of them were differentially expressed in at least one GIT tumor.

**Table 3.** Clinically Important Target-SNPs With Evidence of Disease Association^a^

Target	Target-SNP	Disease	MiRNA	MiRNA Alteration in GIT Cancers; "Expression or Association"	Reference
***BID***	rs8190315	Breast cancer	*hsa-miR-92a-3p*	CRC (down), GC (up)	(35)
***ERCC1***	rs3212986	Multiple cancers	*hsa-miR-92a-3p*	CRC (down), GC (up)	(36)
***ITPA***	rs1127354	Ribavirin-induced anemia and outcomes of therapy in patients infected with HCV	*hsa-miR-93-5p*	GC (up)	(37, 38)
***ZIC2***	rs13542	Orofacial clefts	*hsa-miR-320a*	CRC (up), GC (up)	(39)
***BET1L***	rs2280543	Intracranial aneurysm, uterine fibroids	*hsa-miR-320a*	CRC (up), GC (up)	(40, 41)
***MAPRE1***	rs7270085	Attention deficit hyperactivity disorder (combined symptoms)	*hsa-miR-92a-3p*	CRC (up/down), GC (up)	(42)
***HDAC7***	rs75365750	Obesity-related traits	*hsa-miR-149-5p*	GC (association)	(43)
***ESPL1***	rs1318648	Chronic lymphocytic leukemia	*hsa-miR-149-5p*	GC (association)	(44)
***WWP2***	rs3748386	Myocardial infarction	*hsa-let-7a-5p*	CRC (up)	Genetic Association Database
***DLD***	rs4518	Cardiovascular diseases, P-selectin	*hsa-miR-99a-5p*	EC (down)	(45)

^a^ Abbreviations: CRC, colorectal cancer; Down: downregulated; EC, esophageal cancer; GC, gastric cancer; and Up, upregulated

Consequently, the primary list of 7763 pairs were reduced to 4856 pairs (78 miRNAs and 2745 target genes), which totally hosted 55 miR-SNPs (45 with frequency information) and 6562 target-SNPs (2857 with frequency information).

**Table 4.** Annotation of Twenty Target-SNPs as Expression Quantitative Trait Loci^a,b^

Target Gene	Target-SNP	miRNA	Group	eQTL	TF ^a^ Binding	TF Motif	DNase Footprint	DNase Peak
***HIST1H2AL***	rs200981	*hsa-miR-10b-5p*	B	+	+	+	+	+
***HIST1H2AL***	rs41448545	*hsa-miR-10b-5p*	B	+	+	+	+	+
***ARCN1***	rs17742	*hsa-miR-92a-3p*	B	+	+	+	+	+
***RPS16***	rs17626	*hsa-miR-17-3p*	B	+	+	+	+	+
***H1F0***	rs6000898	*hsa-let-7a-5p*	B	+	+	+	-	+
***MCM7***	rs2307355	*hsa-miR-503-5p*	B	+	+	-	-	+
***RPL27A***	rs6735	*hsa-miR-342-3p*	B	+	+	-	-	+
***GAPDH***	rs1803621	*hsa-miR-149-5p*	B	+	+	-	-	+
***TUBA1B***	rs2753	*hsa-miR-320a*	B	+	+	-	-	+
***GCN1L1***	rs2286050	*hsa-miR-423-3p*	B	+	+	-	-	+
***KIF23***	rs11852675	*hsa-miR-25-3p*	B	+	+	-	-	+
***MYO1D***	rs2285428	*hsa-miR-320a*	B	+	+	-	-	+
***ACTG1***	rs11549223	*hsa-miR-221-3p*	B	+	+	-	-	+
***NR2F6***	rs2288539	*hsa-miR-196a-5p*	B	+	+	-	-	+
***XPO4***	rs4617691	*hsa-miR-18a-3p*	B	+	+	-	-	-
***PEX6***	rs1129187	*hsa-miR-149-5p*	B	+	-	-	-	+
***SOD2***	rs7855	*hsa-miR-769-3p*	C	+	-	-	-	+
***PDHX***	rs497582	*hsa-miR-181b-5p*	B	+	-	-	-	+
***WWP2***	rs3748386	*hsa-let-7a-5p*	B	+	-	-	-	+
***MYO19***	rs2306590	*hsa-miR-615-3p*	C	+	-	-	-	+

^a^ Abbreviations: eQTL, expression quantitative trait loci; and TF, transcription factor.

^b^ The overlap of target-SNPs with different genomic features has been indicated with the “+” symbol, whereas “-” indicates no overlap. Note that some SNPs are located in coding region or 5' UTR.

To gain further insights into the potential functional relevance of target-SNPs, we searched the dbGaP (46), NHGRI GWAS catalog (47), and Genetic Association Database (GAD) (48) to extract information on their association with diseases. Interestingly, association of eleven SNPs with diseases were previously reported (Table 3) (37-45). Among these SNPs, rs8190315 and rs3212986 are located in *BID* and *ERCC1*, respectively. These two regions are recognized by *hsa-miR-92a-3p*, a miRNA that is downregulated in CRC and upregulated in GC.

### 4.3. Group-C (Non-GI-miRNA:GI-Gene Pairs)

MiRNAs that regulate GI-genes were assigned to this group, which contains 214 unique pairs (68 miRNAs and 107 target genes). We identified 45 miR-SNPs (37 with frequency information) and 294 target-SNPs (133 with frequency information).

### 4.4. Annotation of Target-SNPs

Using RegulomeDB, we annotated target-SNPs in groups A, B, and C. A total of 815 target-SNPs overlap with transcription factor binding sites and DNase I hypersensitive regions. Out of these 815 target-SNPs, 24 reside in transcription factor motifs and/or footprints. Furthermore, we found 20 target-SNPs that function as eQTLs (Table 4).

## 5. Discussion

Increasing evidences propose that SNPs within miRNAs and their 3' UTR binding sites may play active roles in a variety of human diseases, especially GIT cancers. Here, we catalogued miRNA-target gene pairs with varying levels of implications for esophageal, gastric and colorectal tumors, and annotated the presence of potentially functional SNPs. We obtained a list of more than 5100 GIT cancers-related miRNA-target gene pairs, hosting 91 miR-SNPs and 7006 target-SNPs, and prioritized them according to experimental findings. 

In group-A, we demonstrated several novel GIT cancers-related interactions and made a list of highly polymorphic pairs. MiR-SNP can alter the expression level of the wild-type miRNA, whereas the presence of a functional SNP in the 3' UTR target site may potentially affect the binding with a specific miRNAs and alter the posttranscriptional regulation by miRNAs. Therefore, we concluded that it would be a good practice to examine the effects generated by the presence of miR-SNPs and target-SNPs in highly polymorphic pairs when studying their contribution to the cancers development or evaluating the predisposition to it. Overall, 97 miRNA-target pairs and their 86 SNPs identified in this group represent prioritized candidate pairs to be considered in further experimental studies for their high probability of involvement in an impaired modulation of gene expression. 

Regarding pairs in group-B, we showed that a considerable proportion of target genes are involved in the biological processes altered in GIT cancers and are differentially expressed in these tumors. Incidentally, we further explored the functional roles of some of these variants by indicating that 11 SNPs with strong evidence of association with a variety of diseases ranging from breast cancer to orofacial cleft actually are located inside binding sites of clinically important miRNAs. The most noteworthy SNP may be rs3212986 in *ERCC1* gene with reported association with multiple cancer types including, but not limited to, estrogen-related cancers (breast, cervical, and ovarian), smoking-related cancers (lung, esophageal, bladder, head and neck, and pancreatic cancer), and brain tumors. Therefore, a large number of pairs and polymorphisms found in group-B, which were not explored in this work, could represent a further line of research in a near future. 

Regarding group-C, our results showed that several miRNAs regulating GI-genes could be potentially relevant and could offer the opportunity to detect other novel candidate miRNAs. Since variations in genomic regulatory regions have crucial functional effects, we exploited the information of the ENCODE project to classify the target-SNPs obtained in our work. Table 4 presents twenty target-SNPs in the context of ENCODE.

In summary, our data provided a comprehensive source of prioritized and annotated candidate polymorphisms within miRNAs sequences and their 3' UTR target sites in GIT cancers, which could facilitate the process of selecting the right candidate miRNA-target genes for functional studies and focusing on potentially relevant polymorphisms for further association studies.
